# Supplementary material for: Efficient Large-Scale and Scarless Genome Engineering Enables the Construction and Screening of Bacillus subtilis Biofuel Overproducers
Source: Int J Mol Sci. 2022 Apr 27;23(9):4853. doi: 10.3390/ijms23094853 (PMC9099979; doi:10.3390/ijms23094853)
Supplement: Supplementary file 1 [file ijms-23-04853-s001.zip › ijms-1685330-supplementary.pdf]

Efficient large-scale and scarless genome engineering enables the construction and screening of *Bacillus subtilis* biofuel overproducers

Jiheng Tian, Baowen Xing, Mengyuan Li, Changgeng Xu, Yi-Xin Huo*, Shuyuan Guo*

Supplementary Materials

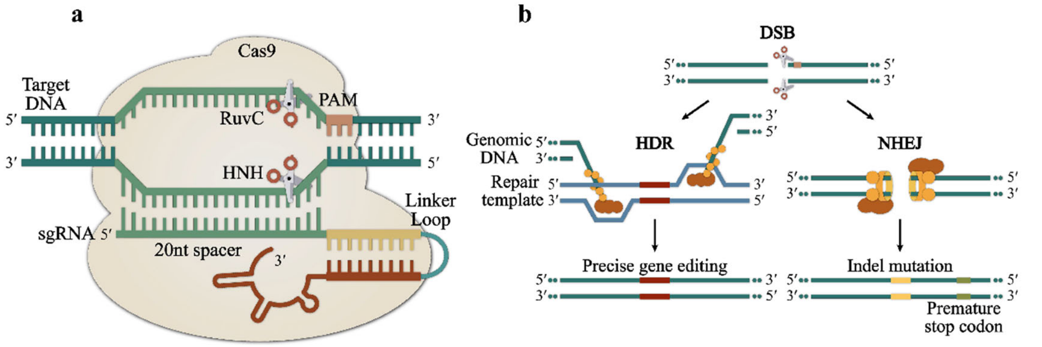

Figure S1 Mechanism of CRISPR-Cas9–mediated genome targeting.

(a) TracrRNA and crRNA were designed as a single guide RNA (sgRNA). At the 5' end of the sgRNA, the 20-nucleotide sequence which is adjacent to a PAM determines the target DNA site by base pairing. And at the 3' end of the guide sequence binds to Cas9 protein. The HNH domain of Cas9 is responsible for cleaving the DNA strand that is complementary to the 20-nucleotide sequence of the sgRNA. The RuvC-like domain of Cas9 cleaves the DNA strand opposite the complementary strand.

(b) DNA double-strand breaks (DSBs) induced by Cas9 cleavage can be repaired by error-prone nonhomologous end-joining (NHEJ) or precise homology-directed repair (HDR).

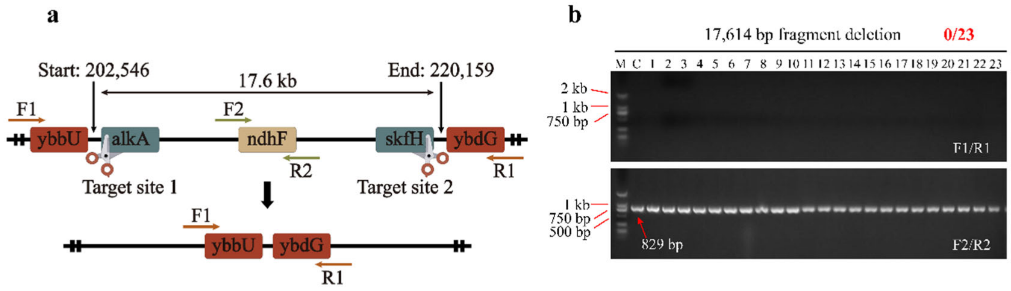

Figure S2. Schematic of the 17.6 kb long fragment deletions by NHEJ system.

(a) Deletion of 17.6 kb genomic fragment in *B. subtilis*.

(b) Representative PCR results of a 17.6 kb fragment deletion. Colonies were randomly picked for PCR screening, and a wild-type colony served as control (abbreviated to C). Primers F1/R1 and F2/R2 were used to prove the fragment was successfully deleted. And if so, the F1/R1 would have positive PCR result whereas F2/R2 would not obtain any result.

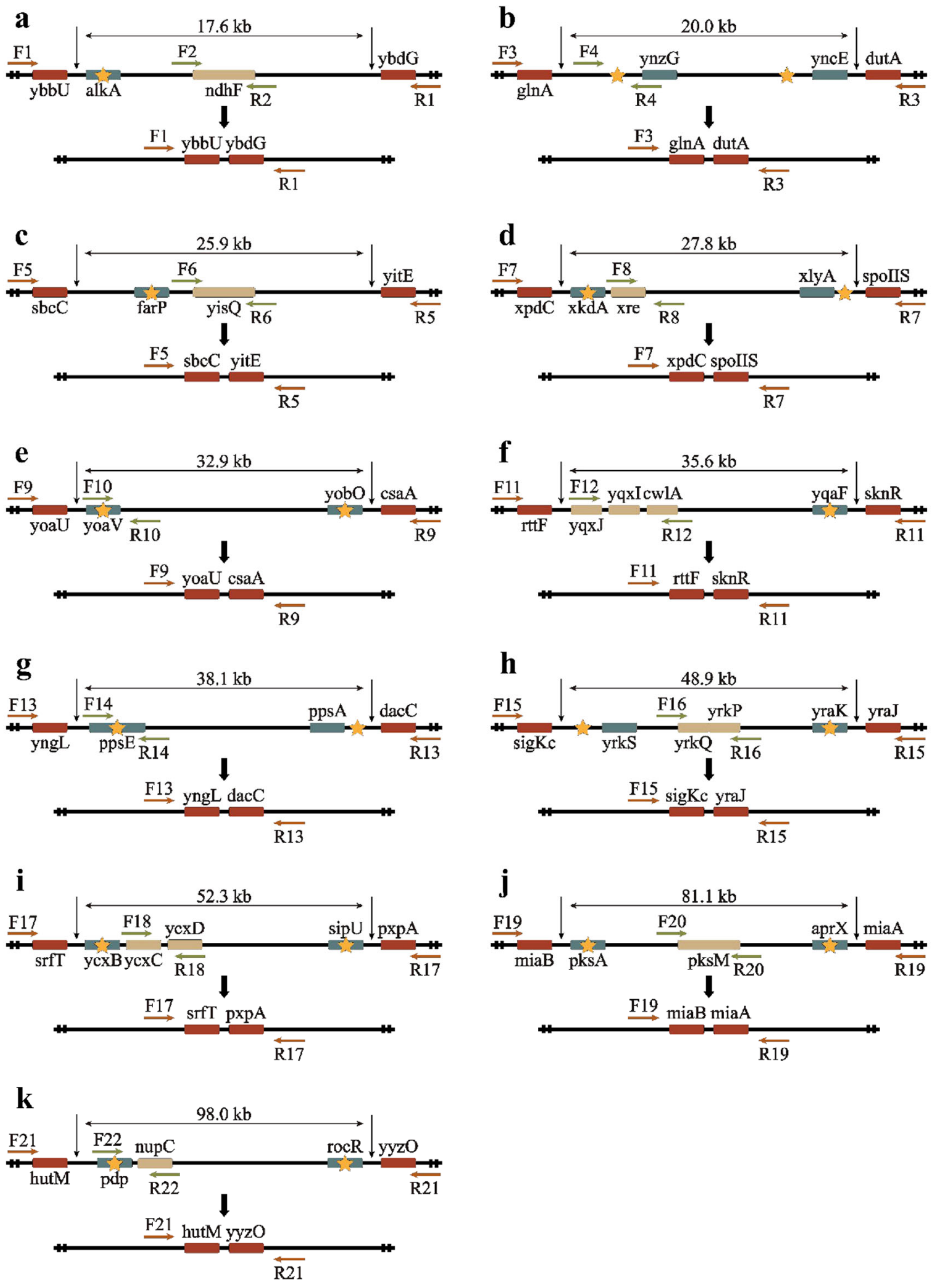

Figure S3. Schematic of different long fragment deletions. (a) Deletion of the 17.6 kb fragment. **(b)** Deletion of the 20.0 kb fragment. **(c)** Deletion of the 25.9 kb fragment. **(d)** Deletion of the 27.8 kb fragment. **(e)** Deletion of the 32.9 kb fragment. **(f)** Deletion of the 35.6 kb fragment. **(g)** Deletion of the 38.1 kb fragment. **(h)** Deletion of the 48.9 kb fragment. **(i)** Deletion of the 52.3 kb fragment. **(j)** Deletion of the 81.1 kb fragment. **(k)** Deletion of the 98.0 kb fragment.

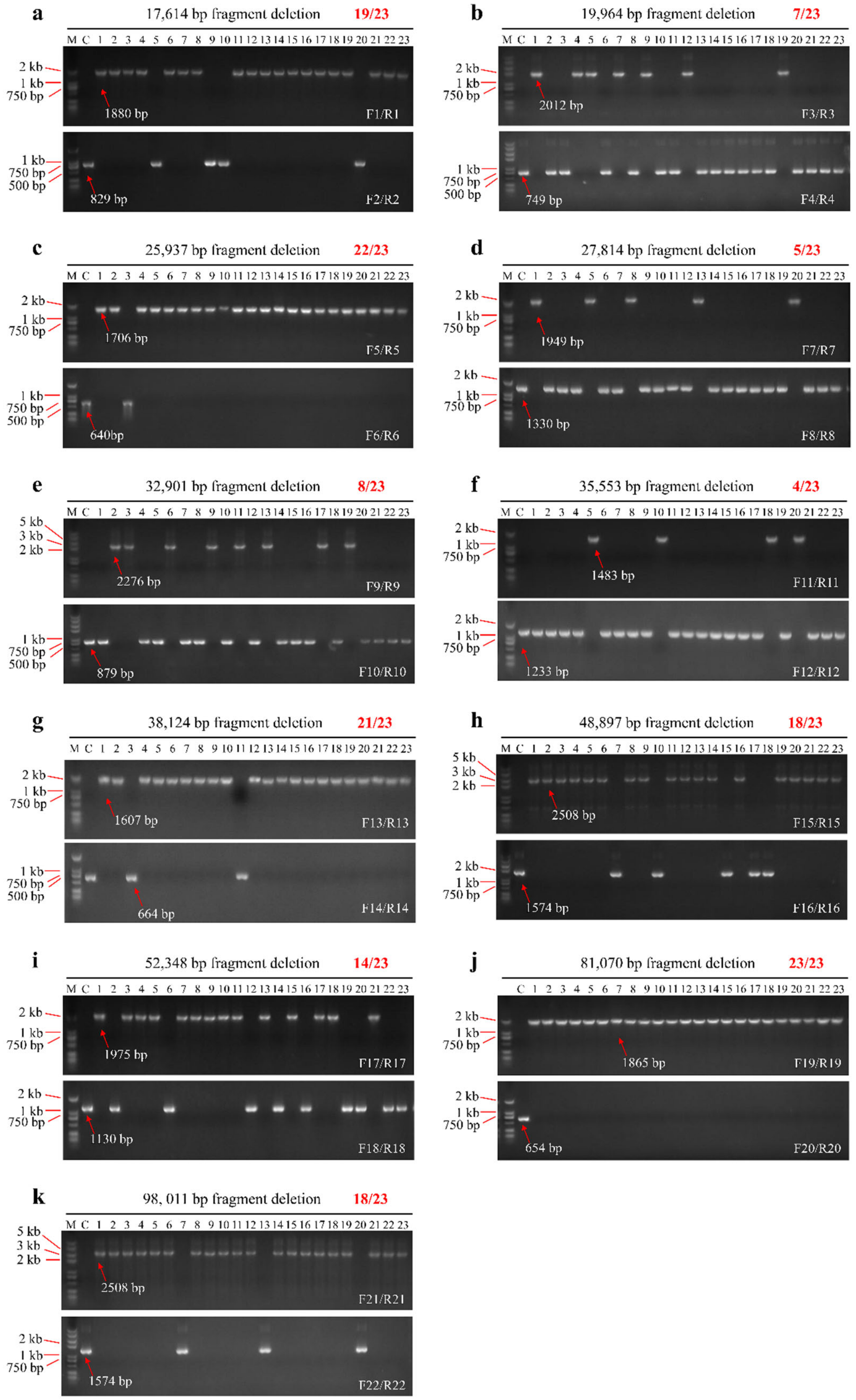

a
17,614 bp fragment deletion
19/23
1880 bp
F1/R1
829 bp
F2/R2
b
19,964 bp fragment deletion
7/23
2012 bp
F3/R3
749 bp
F4/R4
c
25,937 bp fragment deletion
22/23
1706 bp
F5/R5
640bp
F6/R6
d
27,814 bp fragment deletion
5/23
1949 bp
F7/R7
1330 bp
F8/R8
e
32,901 bp fragment deletion
8/23
2276 bp
F9/R9
879 bp
F10/R10
f
35,553 bp fragment deletion
4/23
1483 bp
F11/R11
1233 bp
F12/R12
g
38,124 bp fragment deletion
21/23
1607 bp
F13/R13
664 bp
F14/R14
h
48,897 bp fragment deletion
18/23
2508 bp
F15/R15
1574 bp
F16/R16
i
52,348 bp fragment deletion
14/23
1975 bp
F17/R17
1130 bp
F18/R18
j
81,070 bp fragment deletion
23/23
1865 bp
F19/R19
654 bp
F20/R20
k
98, 011 bp fragment deletion
18/23
2508 bp
F21/R21
1574 bp
F22/R22

Figure S4. Representative results of PCR verification in large fragments deletion experiments. (a) PCR verification of fragment 17.6 kb deletion. **(b)** PCR verification of fragment 20.0 kb deletion. **(c)** PCR verification of fragment 25.9 kb deletion. **(d)** PCR verification of fragment 27.8 kb deletion. **(e)** PCR verification of fragment 32.9 kb deletion. **(f)** PCR verification of fragment 35.6 kb deletion. **(g)** PCR verification of fragment 38.1 kb deletion. **(h)** PCR verification of fragment 48.9 kb deletion. **(i)** PCR verification of fragment 52.3 kb deletion. **(j)** PCR verification of fragment 81.1 kb deletion. **(k)** PCR verification of fragment 98.0 kb deletion.

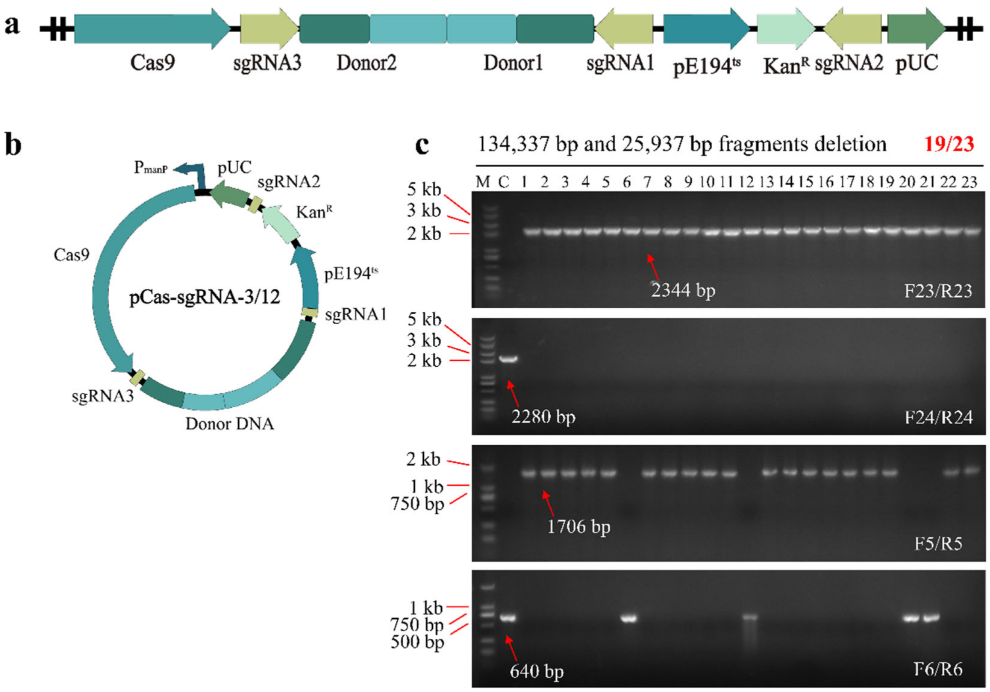

Figure S5. Design of multiplex fragment deletions

(a) Gene elements on plasmid pCas-sgRNA-3/12. Among them, sgRNA1 and sgRNA2 target the ends of the 134.3 kb fragment, while sgRNA3 targets the middle of the 25.9 kb fragment.

(b) The map of plasmid pCas-sgRNA-3/12, which was used to delete both 25.9 and 134.3 kb fragments.

(c) PCR verification of 25.9 and 134.3 kb fragments deletion. The fragment deletions of 134.3 and 25.9 kb were identified using primers F23/R23 and F5/R5, respectively, outside the homology arm. The results showed that the 134.3 kb fragment deletion in colonies 6, 12, 20 and 21 was successful, while the 25.9 kb fragment was not deleted. The remaining 19 single colonies with two fragments deleted simultaneously were successful with a positivity rate of 82.6%.

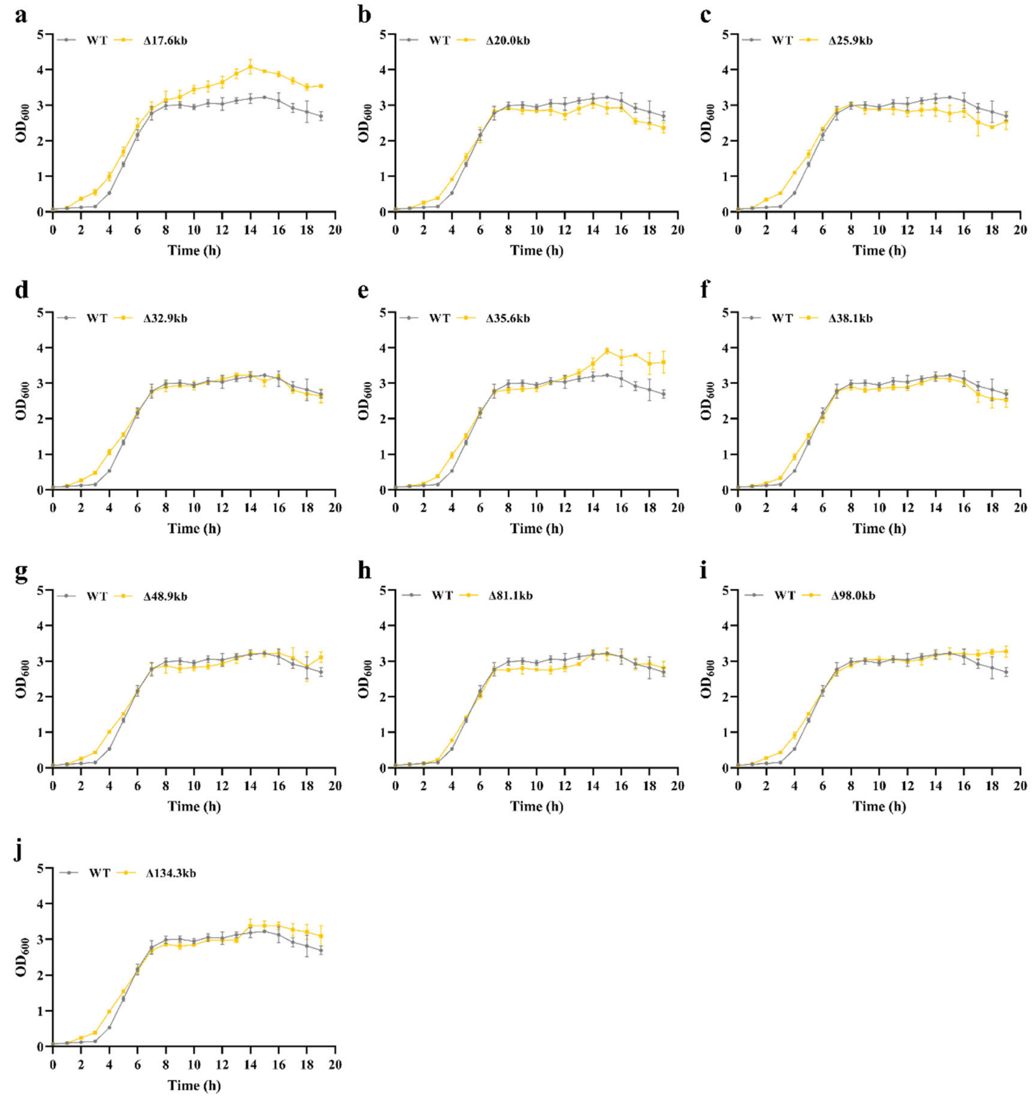

Figure S6. Comparison of growth curves between wild-type and large fragment deletion strains. (a) Growth curve of strain Δ17.6 kb (Guo1). **(b)** Growth curve of strain Δ20.0 kb (Guo2). **(c)** Growth curve of strain Δ25.9 kb (Guo3). **(d)** Growth curve of strain Δ32.9 kb (Guo5). **(e)** Growth curve of strain Δ35.6 kb (Guo6). **(f)** Growth curve of strain Δ38.1 kb (Guo7). **(g)** Growth curve of strain Δ48.9 kb (Guo8). **(h)** Growth curve of strain Δ81.1 kb (Guo10). **(i)** Growth curve of strain Δ98.0 kb (Guo11). **(j)** Growth curve of strain Δ134.3 kb (Guo12).

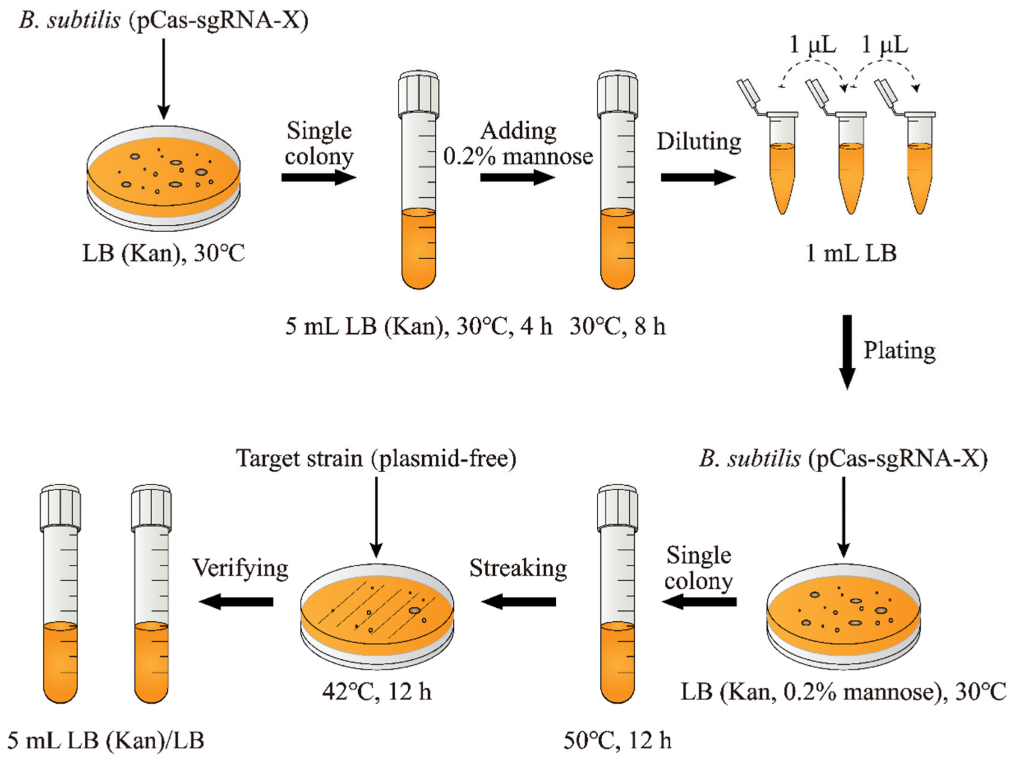

Figure S7. Genome editing and plasmid curing procedure.

The plasmid was transformed into *B. subtilis* by chemical transformation method and incubated on LB agar plate containing kanamycin at 30°C. The next day, single colonies were selected for enrichment culture. When OD_{600}= 0.4-0.6, mannose was added to induce the transcription of Cas9 under the control of P_{manP}. After continuous dilution, the bacteria solution was incubated on LB agar plates containing kanamycin and mannose at 30°C. After editing, single colony was picked and inocubated at 50°C in LB medium without antibiotics. On the next day, the strains were streaked on LB plates to obtain single colony at 42°C. For further verification, single colony was separately inoculated into LB medium with and without kanamycin.

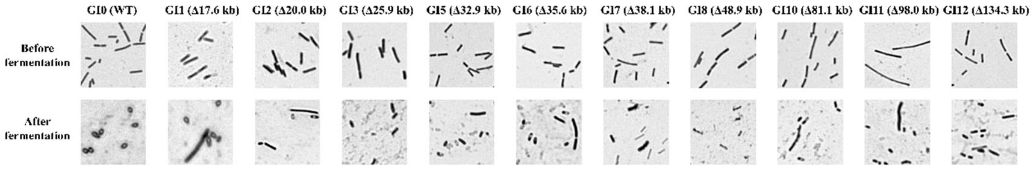

Figure S8. Comparison of the morphology of the wild-type strain GI0 and the deletion mutant before and after 72 h fermentation.

After 72 hours of microaerobic fermentation in shake flasks, the bacterial morphology changed. Almost all of the wild-type strain GI0 formed spores. Some strains GI1 and GI2 also formed spores. The remaining deletion mutants did not form spores, but the bacterial morphology became shorter.

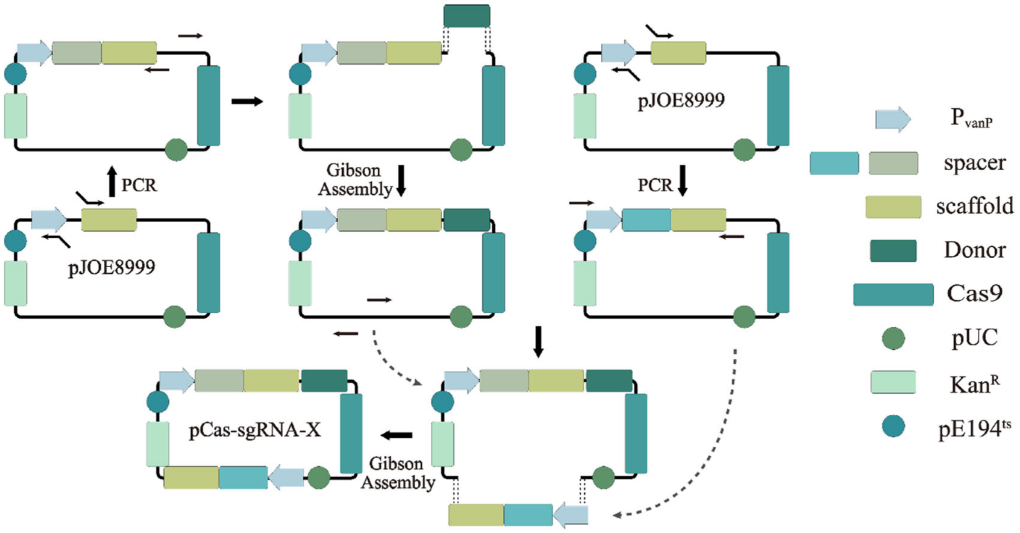

Figure S9. Construction procedures of plasmid pCas-sgRNA-X.

The pUC minimal origin was used for plasmid replication in *E. coli*, the temperature-sensitive replication origin of plasmid $pE194^{ts}$ was used in *B. subtilis*, and a kanamycin resistance gene working in both organisms. In addition, it carries the *cas9* gene under the control of the *B. subtilis* mannose-inducible promoter P_{manP} and a sgRNA-encoding sequence transcribed via a strong constitutive promoter. A high-scoring 20-nucleotide (nt) sequence (found in the website, CHOPCHOP: http://chopchop.cbu.uib.no/, 31 March 2022) can be directly introduced into plasmid by primer. Donor DNA were amplified by PCR from *B. subtilis* 168 chromosomal DNA. Finally, the second 20nt was assembled into the plasmid by Gibson Assembly, and the double sgRNA plasmid was obtained.

Table S1. Strains used in this study

Strain	Genotype or description	Source/Reference
E. coli strain		
JM109	*mcrA recA1 supE44 endA1 hsdR17 gyrA96 relA1 thi Δ(lac-proAB) F' [traD36 proAB+ lacI q lacZDM15]*	Laboratory stock
B. subtilis strain		
B. subtilis 168	Tryptophan auxotrophic (*trpC2*)	Laboratory stock
GN1	The *nprB* gene (1,186,397–1,186,892) was deleted from the genome	This study
GN2	The *vpr* gene (3,908,811–3,909,300) was deleted from the GN1	This study
Guo1	A 17.6 kb fragment (202,546–220,159) was deleted from the genome	This study
Guo2	A 20.0 kb fragment (1,880,087–1,900,050) was deleted from the genome	This study
Guo3	A 25.9 kb fragment (1,148,155–1,174,091) was deleted from the genome	This study
Guo4	A 27.8 kb fragment (1,320,486–1,348,299) was deleted from the genome	This study
Guo5	A 32.9 kb fragment (2,045,929–2,078,829) was deleted from the genome	This study
Guo6	A 35.6 kb fragment (2,663,328–2,698,880) was deleted from the genome	This study
Guo7	A 38.1 kb fragment (1,960,139–1,998,262) was deleted from the genome	This study
Guo8	A 48.9 kb fragment (2,701,779–2,750,675) was deleted from the genome	This study
Guo9	A 52.3 kb fragment (404,400–456,747) was deleted from the genome	This study
Guo10	An 81.1 kb fragment (1,781,781–1,862,850) was deleted from the genome	This study
Guo11	A 98.0 kb fragment (4,049,079–4,147,089) was deleted from the genome	This study
Guo12	A 134.3 kb fragment (2,152,114–2,286,450) was deleted from the genome	This study
Guo13	The 17.6, 20.0, 38.1 and 98.0 kb fragments were deleted from the Guo11	This study
Guo14	The 25.9 and 134.3 kb fragments were deleted from the Guo13	This study
Guo15	*B. subtilis* 168 (Δ134.3 kb::P_{grac}-purple)	This study
GI0	*B. subtilis* 168 with pCas-69 and pHT01-65	This study
GI1	Guo1 with pCas-69 and pHT01-65	This study
GI2	Guo2 with pCas-69 and pHT01-65	This study
GI3	Guo3 with pCas-69 and pHT01-65	This study
GI5	Guo5 with pCas-69 and pHT01-65	This study
GI6	Guo6 with pCas-69 and pHT01-65	This study
GI7	Guo7 with pCas-69 and pHT01-65	This study
GI8	Guo8 with pCas-69 and pHT01-65	This study
GI10	Guo10 with pCas-69 and pHT01-65	This study
GI11	Guo11 with pCas-69 and pHT01-65	This study
GI12	Guo12 with pCas-69 and pHT01-65	This study

Table S2. Reagents and media used in this study

Reagent	Formulation concentration	Working concentration in *E. coli*	Working concentration in *B. subtilis*
Ampicillin	100 g/L	0.1 g/L	
Chloramphenicol	25 g/L	0.025 g/L	0.01 g/L
Kanamycin	50 g/L	0.05 g/L	0.02 g/L
Glucose	500 g/L		10 g/L
IPTG	1 mol		1 mmol
D-mannose	0.2 g/mL		2 mg/mL

Medium	Composition
LB	10 g/L tryptone, 5 g/L yeast extract, 10 g/L NaCl; Solid medium (20 g/L agar)
GM1	0.2% ammonium sulfate, 1.4% dipotassium hydrogen phosphate, 0.6% potassium dihydrogen phosphate, 0.07% sodium citrate, 0.5% glucose, 0.02% magnesium sulfate heptahydrate, 0.2% yeast extract, and 0.025% casamino acids
GM2	0.2% ammonium sulfate, 1.4% dipotassium hydrogen phosphate, 0.6% potassium dihydrogen phosphate, 0.07% sodium citrate, 0.5% glucose, 0.08% magnesium sulfate heptahydrate, 0.1% yeast extract, 0.01% casamino acids, and 0.05% calcium chloride.
LBGSM-I	10 g/L tryptone, 5 g/L yeast extract, 5 g/L NaCl, 20 g/L glucose, 2 g/L K_2HPO_4, 1 g/L KH_2PO_4, 1,000th dilution of Trace Metal Mix A5 (2.86 g H_3BO_3, 1.81 g $MnCl_2{\cdot}4H_2O$, 0.222 g $ZnSO_4{\cdot}7H_2O$, 0.39 g $Na_2MoO_4{\cdot}2H_2O$, 0.079 g $CuSO_4{\cdot}5H_2O$, 49.4 mg $Co(NO_3)_2{\cdot}6H_2O$ per litre water)

Table S3. Plasmids involved in this study

Plasmid	Description	Source/ Reference
pJOE8999	P_{manP}-*cas9*, pUC, pE194^{ts}, *kan^{r}*	Altenbuhner 2016 [1]
pCas-sgRNA-*nprB*	Plasmid used to delete the *nprB* gene (1,186,037–1,187,653)	This study
pCas-sgRNA-*vpr*	Plasmid used to delete the *vpr* gene (3,907,844–3,910,264)	This study
pCas-sgRNA-1	Plasmid used to delete the 17.6 kb fragment (202,546–220,159)	This study
pCas-sgRNA-2	Plasmid used to delete the 20.0 kb fragment (1,880,087–1,900,050)	This study
pCas-sgRNA-3	Plasmid used to delete the 25.9 kb fragment (1,148,155–1,174,091)	This study
pCas-sgRNA-4	Plasmid used to delete the 27.8 kb fragment (1,320,486–1,348,299)	This study
pCas-sgRNA-5	Plasmid used to delete the 32.9 kb fragment (2,045,929–2,078,829)	This study
pCas-sgRNA-6	Plasmid used to delete the 35.6 kb fragment (2,663,328–2,698,880)	This study
pCas-sgRNA-7	Plasmid used to delete the 38.1 kb fragment (1,960,139–1,998,262)	This study
pCas-sgRNA-8	Plasmid used to delete the 48.9 kb fragment (2,701,779–2,750,675)	This study
pCas-sgRNA-9	Plasmid used to delete the 52.3 kb fragment (404,400–456,747)	This study
pCas-sgRNA-10	Plasmid used to delete the 81.1 kb fragment (1,781,781–1,862,850)	This study
pCas-sgRNA-11	Plasmid used to delete the 98.0 kb fragment (4,049,079–4,147,089)	This study
pCas-sgRNA-12	Plasmid used to delete the 134.3 kb fragment (2,152,114–2,286,450)	This study
pCas-sgRNA-3/12	Plasmid used to delete the 134.3 kb fragment (2,152,114–2,286,450) and the 25.9 kb fragment (1,148,155–1,174,091)	This study
pCas-12-2000	pCas-sgRNA-12 derivative, containing 2000 bp donor DNA	This study
pCas-12-1500	pCas-sgRNA-12 derivative, containing 1500 bp donor DNA	This study
pCas-12-1000	pCas-sgRNA-12 derivative, containing 1000 bp donor DNA	This study
pCas-12-600	pCas-sgRNA-12 derivative, containing 600 bp donor DNA	This study
pCas-12-360	pCas-sgRNA-12 derivative, containing 360 bp donor DNA	This study
pCas-12-purple	P_{manP}-*cas9*, P_{grac}-*purple,* pUC, pE194^{ts}, *kan^{r}*	This study
pCas-69	P_{manP}-*alsS-ilvC-ilvD*, pUC, pE194^{ts}, *kan^{r}*	This study
pHT01	P_{grac}, pBR322, repA, *amp^{r}*, *cm^{r}*	Laboratory stock
pHT01-purple	P_{grac}-*purple*, pBR322, repA, *amp^{r}*, *cm^{r}*	This study
pHT01-Mtb-NHEJ	P_{grac}-*ligD-mku*, pBR322, repA, *amp^{r}*, *cm^{r}*	This study
pHT01-65	P_{grac}-*kivD-adhA*, pBR322, repA, *amp^{r}*, *cm^{r}*	This study

Table S4. The target sequences (N20 + PAM) designed in this study

Target site 1	Nucleotide sequence (5'-3')	Target site 2	Nucleotide sequence (5'-3')
Δ*nprB*	CTGAACCTGAGCCTGGCTATTGG		
Δ*vpr*	ACGAGGCAGCATTGCATTTGTGG		
NHEJ-Δ17.6 kb1	ATAAGACCCACATCATCGATTGG	NHEJ-Δ17.6 kb2	CGTTTGTCGCACATACAAGACGG
Δ17.6 kb	ATAAGACCCACATCATCGATTGG		
Δ20.0 kb-1	CCAAATGACCATTTCCATAGAGG	Δ20.0 kb-2	TCACAAAAATCGTTTAAGTAAGG
Δ25.9 kb	ATGTACGCATATCGGACGGGCGG		
Δ27.8 kb-1	CAAGCGGCCAAATGCGTACGAGG	Δ27.8 kb-2	CGGGTGAGGGAGCGCAGTGCTGG
Δ32.9 kb-1	CACTTATTTGGGGTTATACTTGG	Δ32.9 kb-2	CTCTAACCGTCATCAAATAGTGG
Δ35.6 kb	GGTGAAAAATGATGAGAAAATGG		
Δ38.1 kb-1	TATCCTCTTATTATGAGAACTGG	Δ38.1 kb-2	GCTCATAAAGACATGCTGGAAGG
Δ48.9 kb-1	TTGACTTATAATCCTCCCGCAGG	Δ48.9 kb-2	AGGGCGTTCGCGCAACGATAAGG
Δ52.3 kb-1	TTCAGGGTCAATACATCAAGTGG	Δ27.8 kb-2	AACAGGTGACTTCGAAGTTGAGG
Δ81.1 kb-1	GCGCCATTATTTCTCTACACAGG	Δ81.1 kb-2	AAGCAAGGAGTCCATTTGAAGGG
Δ98.0 kb-1	TAACGTCGGCCGTCAAACAATGG	Δ98.0 kb-2	TGCGGTATTGATAGGGCAGATGG
Δ134.3 kb-1	TTATTTAAACAAGCTTTGCGGGG	Δ134.3 kb-2	AATGCAAAGAATGAGCTCAATGG

Table S5. Verification primers used in genomic editing experiments

Forward primer	Sequence (5'-3')	Reverse primer	Sequence (5'-3')	PCR product
nprB-F	AGGCTACTTTTTCTTGAATTCCGGTTTG	*nprB*-R	TATGTTAAAGCCCGATAGTAAATTTGTTCACTTG	1764/1268 bp
vpr-F	ACTGGGGTTGAATACAATCACCCA	*vpr*-R	TTTTGTTTCGTTTCCGTTTTCCTTCAAG	1290/800 bp
Δ17.6 kb-F1	GCGACCAGAAAGACGAAATGGAAAT	Δ17.6 kb-R1	ATGTCTATTTTGGTCAGAGACTGCA	1880 bp
Δ17.6 kb-F2	TAAGGTTAAACAGTGCTTGGCAAGT	Δ17.6 kb-R2	GCTCCTCCTAAAACAGTTAAACCGG	829 bp
Δ20.0 kb-F3	GTTCGGTGTAAACGGTTCAGGTATG	Δ20.0 kb-R3	ACGTCTATTTATTCCCTGGCGACAT	2012 bp
Δ20.0 kb-F4	CGCCATGTTTCTAGGATACCGGAAT	Δ20.0 kb-R4	TAAGGAAACCCAATCCTTACTGCGC	749 bp
Δ25.9 kb-F5	GGACTGAGGAGGTTGAACAAAGTCT	Δ25.9 kb-R5	GCCTTCAAAAGGAATGGTCCACATC	1706 bp
Δ25.9 kb-F6	ATCATTGCCATGTAAACCGGGAATT	Δ25.9 kb-R6	TGATTTTCGGTTTGTTCGGCTTTCC	640 bp
Δ27.8 kb-F7	CGCAATTTTGGGTGCCGTGATAAAA	Δ27.8 kb-R7	CGTTTCGAAGCGAAGGTCAAAGAAA	1949 bp
Δ27.8 kb-F8	CGTCATCGGATTTTTTGTCTTTCCC	Δ27.8 kb-R8	CTCTTCTCAGCTCGTCCAAAATCTG	1330 bp
Δ32.9 kb-F9	ACATGGAGTCTCGTACACCTTGGAAAGGTAAGTT	Δ32.9 kb-R9	CTGGCAGAAATCGAGCATTATCACCCAGC	2276 bp
Δ32.9kb-F10	ATGGAATGCAGTTTGTCGATTCAGG	Δ32.9 kb-R10	TTGACAAAAGATCCAGATTGCGGAG	879 bp
Δ35.6 kb-F11	AACCATGGGATATGGGTCAT	Δ35.6 kb-R1	ATTTTTTTCACATACACTTTGCAAGG	1483 bp
Δ35.6 kb-F12	GACCAGCTTATTATGTGGCTG	Δ35.6 kb-R12	AAAGCACTGGCTGCACTATA	1233 bp
Δ38.1 kb-F13	CCCATTTTTTGCACATCGTC	Δ38.1 kb-R13	CGATTACAATTGAAGGGAGCG	1607 bp
Δ38.1 kb-F14	GCTTCGATGAAATGAATGCTTG	Δ38.1 kb-R14	TTAACACAATCAGTATTGATGATGACTTC	664 bp
Δ48.9 kb-F15	AATGCAGCACCCAATGTTGAAGTTC	Δ48.9 kb-R15	TCCTGATTGTCAATCGTGATTACCG	2508 bp
Δ48.9 kb-F16	ATCAAATACACGACCAAGCTCCTCC	Δ48.9 kb-R16	TATTATGATGCCAGGCATTTCGGGA	1574 bp
Δ52.3 kb-F17	CAGTGTCAGAAAAATTATGGCGCTG	Δ52.3 kb-R17	AGCCGCTTTGAGCTGTTTTCTGATT	1975 bp
Δ52.3 kb-F18	ATCGCTGAAGACAAAGCGGACAATA	Δ52.3 kb-R18	CTCTTTCCCATTTGGGAACGGCATG	1130 bp
Δ81.1 kb-F19	GGAAGCGTATGTGATGCCAAGTATG	Δ81.1 kb-R19	GTGAAAAGCCACCACATATTGATGC	1865bp
Δ81.1 kb-F20	CACTATGAACGAAGCTGAGTTGTTG	Δ81.1 kb-R20	CTTCCGCTGATTGCCAATTTTGAAC	654 bp
Δ98.0 kb-F21	TTTATGATCTCATTGGGAGGCGTCA	Δ98.0 kb-R21	AAAACAACAATGGCCGGGAATCAAT	2508 bp
Δ98.0 kb-F22	GACGGATCGTCAACAATTGAGCTGT	Δ98.0 kb-R22	TCGGTATTTCGTTCCAAGGCATTCT	1574 bp
Δ134.3 kb-F23	TTCCTTCGGACCGGTTGGAT	Δ134.3 kb-R23	GCGGGATTTATCAGCGAGCA	2344 bp
Δ134.3 kb-F24	AGGCCTCTAGTCTCCCCAAC	Δ134.3 kb-R24	GGAAACTTTGGCGTGGGTGT	2280 bp
PP-F	GCAACCGTTTTTTCGGAAGGAA	PP-R	ATTCTTTCCGAGCTTCGTCCAAAA	1143 bp

References

1. Altenbuchner, J. Editing of the Bacillus Subtilis Genome by the Crispr-Cas9 System. *Appl Environ Microbiol* **2016**, 82, 5421-5427, doi:10.1128/aem.01453-16.
